# Proteomics as a Quality Control Tool of Pharmaceutical Probiotic Bacterial Lysate Products

**DOI:** 10.1371/journal.pone.0066682

**Published:** 2013-06-19

**Authors:** Günter Klein, Joost P. Schanstra, Janosch Hoffmann, Harald Mischak, Justyna Siwy, Kurt Zimmermann

**Affiliations:** 1 Institute of Food Quality and Food Safety, University of Veterinary Medicine, Hannover, Germany; 2 Institut National de la Santé et de la Recherche Médicale (INSERM), U1048, Institute of Cardiovascular and Metabolic Disease, Toulouse, France; 3 Université Toulouse III Paul-Sabatier, Toulouse, France; 4 Mosaiques Diagnostics, Hannover, Germany; 5 BHF Glasgow Cardiovascular Research Centre, Institute of Cardiovascular and Medical Sciences, Faculty of Medical, Veterinary and Life Sciences, University of Glasgow, Glasgow, United Kingdom; 6 Charite-Universitatsmedizin Berlin, Berlin, Germany; 7 SymbioPharm GmbH, Herborn, Germany; Institut Pasteur de Lille, France

## Abstract

Probiotic bacteria have a wide range of applications in veterinary and human therapeutics. Inactivated probiotics are complex samples and quality control (QC) should measure as many molecular features as possible. Capillary electrophoresis coupled to mass spectrometry (CE/MS) has been used as a multidimensional and high throughput method for the identification and validation of biomarkers of disease in complex biological samples such as biofluids. In this study we evaluate the suitability of CE/MS to measure the consistency of different lots of the probiotic formulation Pro-Symbioflor which is a bacterial lysate of heat-inactivated *Escherichia coli* and *Enterococcus faecalis*. Over 5000 peptides were detected by CE/MS in 5 different lots of the bacterial lysate and in a sample of culture medium. 71 to 75% of the total peptide content was identical in all lots. This percentage increased to 87–89% when allowing the absence of a peptide in one of the 5 samples. These results, based on over 2000 peptides, suggest high similarity of the 5 different lots. Sequence analysis identified peptides of both *E. coli* and *E. faecalis* and peptides originating from the culture medium, thus confirming the presence of the strains in the formulation. Ontology analysis suggested that the majority of the peptides identified for *E. coli* originated from the cell membrane or the fimbrium, while peptides identified for *E. faecalis* were enriched for peptides originating from the cytoplasm. The bacterial lysate peptides as a whole are recognised as highly conserved molecular patterns by the innate immune system as microbe associated molecular pattern (MAMP). Sequence analysis also identified the presence of soybean, yeast and casein protein fragments that are part of the formulation of the culture medium. In conclusion CE/MS seems an appropriate QC tool to analyze complex biological products such as inactivated probiotic formulations and allows determining the similarity between lots.

## Introduction

Probiotic bacteria have a wide range of applications in veterinary and human therapeutics [1]–[4]. The internationally endorsed definition of probiotics determines probiotics as well-defined micro-organisms that, when obtained in sufficient active quantities in the gut, lead to positive effects on health (like stabilising the gut microflora, reconstitution of the gut flora after antibiotic therapy or having a protective effect against travellers diarrhoe) [5], [6]. Products with bacterial lysates contain heat-inactivated probiotic bacteria. In that sense bacterial lysates are not probiotics *sensu stricto*, but they are produced with probiotic bacteria and can exhibit similar beneficial effects on the host as live bacteria [7]. Most of the proven health effects probiotics elicit are provided in the gastro-intestinal tract (GIT). Probiotics using live bacteria maintain or promote the GIT homeostasis, and probiotics have been found to stimulate the growth of indigenous beneficial gut microbes such as bifidobacteria and inhibit the growth of pathogenic or opportunistic pathogenic microbes [8]–[10]. In addition, inactivated cells (heat or radiation induced) can also improve the health of the host as shown in patients with irritable bowel syndrome (IBS) [11]. In these patients administration of a bacterial lysate of heat-inactivated *Enterococcus faecalis* (*E. faecalis*: DSM16440) and *Escherichia coli* (*E. coli*: DSM17252), which was identical to the bacterial lysate used in our study, significantly improved IBS symptoms compared to placebo [11]. The mechanisms of these inactivated bacteria on IBS are less clear, but *in vitro* studies have shown that the beneficial effects might originate from the anti-inflammatory properties of these inactivated bacteria [7], [12]. A number of bacterial lysates are commercially available including Pro-Symbioflor containing an *E. faecalis and Escherichia coli* lysate [11], [13], Colibiogen containing an *E. coli* lysate [14], CytoFlora® containing lysed cell walls of *Bifidobacterium bifidum*, *Bifidobacterium infantis*, *Bifidobacterium longum*, *Streptococcus thermophilus*, *Lactobacillus acidophilus*, *Lactobacillus rhamnosus*, *Lactobacillus plantarum*, *Lactobacillus salivarius*, *Lactobacillus reuteri*, *Lactobacillus casei*, *Lactobacillus bulgaricus*, and a species called “Lactobacillus sporogenes” (which is not an official species name) [15] and Del-Immune V® containing cell wall peptidoglycans and DNA fragments of *Lactobacillus rhamnosus* [16]. Besides for *E. coli* or *Enterococcus*, *in vitro* studies are also available for *Lactobacillus rhamnosus* or *Streptococcus thermophilus* to support the potential beneficial effects on health [7], [12]. Finally, the field of application seems to have widened recently since bacterial lysates have been shown as an alternative therapy of chronic obstructive pulmonary disease (COPD) [17].

In rare cases probiotic bacteria can be isolated from human clinical samples in nosocomial infections [18]. To be able to identify those cases, the identity of the probiotic strain and its characterisation by molecular methods must be provided by the manufacturer. The concept of “Qualified Presumption of Safety” (QPS) as developed by the European Food Safety Authority (EFSA) consequently requires the identification of probiotic microorganism and also the identification of the organism in the final product [19]. The requirements for quality control (QC) are similar for probiotics with viable bacteria or bacterial lysates. In Europe, this is regulated by European law and in the member states enforced by national legislation (in Germany e.g. by Arzneimittelgesetz, supervised by the national Federal Institute for Drugs and Medicinal Devices) [20]. The difficulty associated with maintaining a high degree of QC is inhibiting the development of probiotic-based therapeutics.

Before bacterial cells of these lysates are autoclaved or irradiated, regular quantitative and qualitative cultural and genotypic QC methods are applied, as is required for viable products [5]. These methods are not applicable for the final product since no cultural methods can be applied and only molecular methods are applicable. However, the complexity of bacterial lysates is high and therefore QC of the lysates is best performed with multidimensional techniques allowing the analysis of a maximum of molecular features at one time. Indirect techniques like proteomics are potentially suitable to describe the molecular features in such complex samples. Proteomics, the analysis of the total protein content of a sample, is a multidimensional technique potentially able to grasp the complexity of bacterial lysates. Proteomics has been used in a descriptive way to determine the differences between a number of potential probiotic bacterial isolates [21], [22]. These studies provide comprehensive catalogues and carry promise for the discovery of novel probiotic effector molecules. However these techniques are labour intensive and reproducibility, if not analysed in the same batch, is in general low.

Capillary electrophoresis coupled to mass spectrometry (CE/MS) is a high resolution and high throughput technology that has been successfully applied for the discovery and validation of peptide biomarkers of disease in biofluids [23]. CE/MS analysis allows detecting thousands of peptides in a biofluid sample. Furthermore, CE/MS has high reproducibility allowing the comparison of the protein content of samples over time [24]. Therefore in the current study we evaluated the suitability of CE/MS to serve as a high throughput technique for the QC analysis of complex samples such as inactivated probiotics.

## Materials and Methods

### Bacterial strains

We used an autolysate of cells and cell fragments of *E. faecalis* (DSM 16440) and *E. coli* (DSM 17252) called Pro-Symbioflor. 1.5 mL of Pro-Symbioflor contains 3.0 to 9.0 10^7^ CFU of living bacteria before inactivation. Rapidly after combining both strains are exposed to heat and pressure (via autoclave), and the final product is sterile thereafter.

Five Pro-Symbioflor lots were investigated in this study. The lots were provided directly from the manufacturer (Symbiopharm GmbH, Herborn, Germany) and stored according to the manufacturers instructions at room temperature until investigation. In addition, for the comparison, a growth medium sample without bacteria was also studied by CE/MS. The composition of this culture medium is listed in **Table 1**. This growth medium is identical to the medium used in commercial production of the product.

**Table 1:** Composition of culture medium.

medium composition	concentration (mg/ml)
lactose monohydrate	4.9000
sodium carbonate decahydrate	0.9600
sodium chloride	3.8000
magnesium sulfate heptahydrate	0.7790
potassium chloride	0.1330
calcium chloride dihydrate	0.0760
magnesium chloride hexahydrate	0.6080
standard nutrient broth 0.020 ml consisting of:	
casein peptone (soybean)	0.300
yeast extract	0.060
sodium chloride	0.120
glucose monohydrate	0.020

### Sample preparation

For the analysis of the peptide content of bacterial lysates, 2.5 ml of Pro-Symbioflor was used. To decrease matrix effects by removing electrolytes and salts, and also to enrich for polypeptides, the samples were loaded onto PD-10 desalting column (GE Healthcare, Sweden) equilibrated with 25 mL 0.01% NH_4_OH in HPLC-grade H_2_O (Roth, Germany). Peptides were eluted by adding 2.5 mL of 0.01% NH_4_OH. Finally, samples were lyophilized and stored at 4°C. Shortly before CE-MS analysis, lyophilized samples were resuspended in HPLC-grade H_2_O.

### Capillary Electrophoresis-Mass Spectrometry (CE/MS) analysis

CE/MS analysis was performed as previously described using a P/ACE MDQ capillary electrophoresis system (Beckman Coulter, Fullerton, USA) on-line coupled to a MicroTOF MS (Bruker Daltonic, Bremen, Germany) [25]. The ESI sprayer (Agilent Technologies, Palo Alto, USA) was grounded, and the ion spray interface potential was set between −4.0 and −4.5 kV. Data acquisition and MS acquisition methods were automatically controlled by the CE via contact-close-relays. Spectra were accumulated every 3 s over a range of m/z 350 to 3000. Details on accuracy, precision, selectivity, sensitivity, reproducibility, and stability of the CE-MS method have been provided previously [24], [26].

### Data processing

Mass spectral ion peaks representing identical molecules at different charge states were deconvoluted into single masses using MosaiquesVisu software [27]. Only those signals with z>1 that were observed in a minimum of 2 consecutive spectra with signal-to-noise ratios >4 were included. The software employs a probabilistic clustering algorithm and uses both isotopic distribution as well as conjugated masses for charge-state determination of peptides/proteins. The resulting peak list characterizes each polypeptide by its molecular mass, CE-migration time, and ion signal intensity (amplitude) value. To minimize effects of biological and analytical variability between the different lots a normalization of retention time, signal intensity and mass was performed. The CE migration time was normalized by local regression using 281 references between 17 and 46 Minutes. The masses were calibrated utilizing 274 reference masses in the range of 800–3000 Da by applying linear regression. Normalization of signal intensities was based on 43 “internal standard” peptides detected in all 5 lots with relative low cV, similarly to what has been described for urinary peptidome analysis [28]. Here linear regression was applied as well.

All detected peptides were deposited, matched, and annotated in a Microsoft SQL database, allowing further analysis and comparison of multiple samples [29]. Peptides were considered identical within different samples, when mass deviation was lower than 50 ppm for small peptides or 75 ppm for larger peptides and proteins. Due to analyte diffusion, CE peak widths increase with CE migration time. In the data clustering process this effect was compensated by linearly increasing cluster widths over the entire measurement from 2 to 5%.

### Peptide sequencing

Bacterial lysates were separated on a Dionex Ultimate 3000 RSLS nano flow system (Dionex, Camberly UK). A 5 ml sample was loaded in 0.1% formic acid and acetonitrile (98∶2) onto a Dionex 100 mm32 cm, 5 mm C18 nano trap column at a flowrate of 5 ml/min. Elution was performed on an Acclaim PepMap C18 nano column 75 mm315 cm, 2 mm, 100 Å with a linear gradient of 0.1% formic acid against 100% acetonitrile starting at 5%–50% over 100 min. The sample was ionised in positive ion mode using a Proxeon nano spray ESI source (Thermo Fisher Hemel UK) and analysed in an Orbitrap Velos FTMS (Thermo Finnigan, Bremen, Germany). The MS was operated in data-dependent mode to switch between MS and MS/MS acquisition and parent ions were fragmented by high-energy collision-induced dissociation. Data files were searched against *E. coli*, *E. faecalis*, *Bos Taurus*, Yeast and Soyabean entries in the Swiss-Prot database without any enzyme specificity using Open Mass Spectrometry Search Algorithm (OMSSA, http://pubchem.ncbi.nlm.nih.gov/omssa) with an e-value cut-off of 0.1. No fixed modification and oxidation of methionine as variable modifications were selected. Mass error windows of 10 ppm and 0.05 Da were allowed for MS and MS/MS, respectively. For further validation of obtained peptide identifications, the strict correlation between peptide charge at pH 2 and CE-migration time was utilized to minimize false-positive identification rates [30]. Calculated CE-migration time of the sequence candidate based on its peptide sequence (number of basic amino acids) was compared to the experimental migration time. Peptides were accepted only if they had a mass deviation below ±80 ppm and a CE-migration time deviations below ±2 min.

## Results

### Comparison of the different bacterial lysate preparations

**Figure 1:**
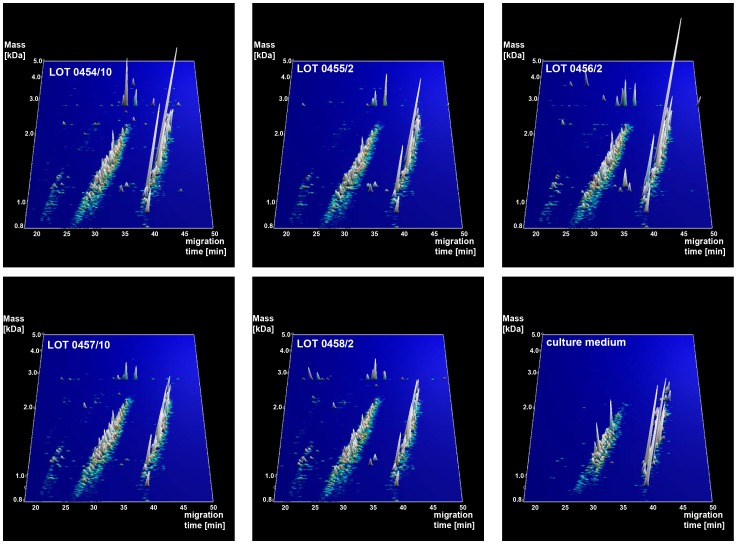
Peptide content of bacterial lysates and medium. Contourplots showing all polypeptides detected in the 5 lots of Pro-SymbioFlor and culture medium, displayed in three dimensions (x-axis: migration time in Minutes, y-axis: mass in logarithmic scale and Da, peak height (z-axis): signal intensity).

Five different lots of the bacterial lysates (lot 0454/10, 0455/2, 0456/2, 0457/10, 0458/2) as well as the culture medium were analyzed by CE/MS. A total of 5217 different peptides could be detected in all lots (**Figure 1**). Each peak in the contourplots represents a peptide that is tagged by a unique identifier consisting of a mass and migration time. The height of each peak indicates the abundance of the different peptides.

**Figure 2:**
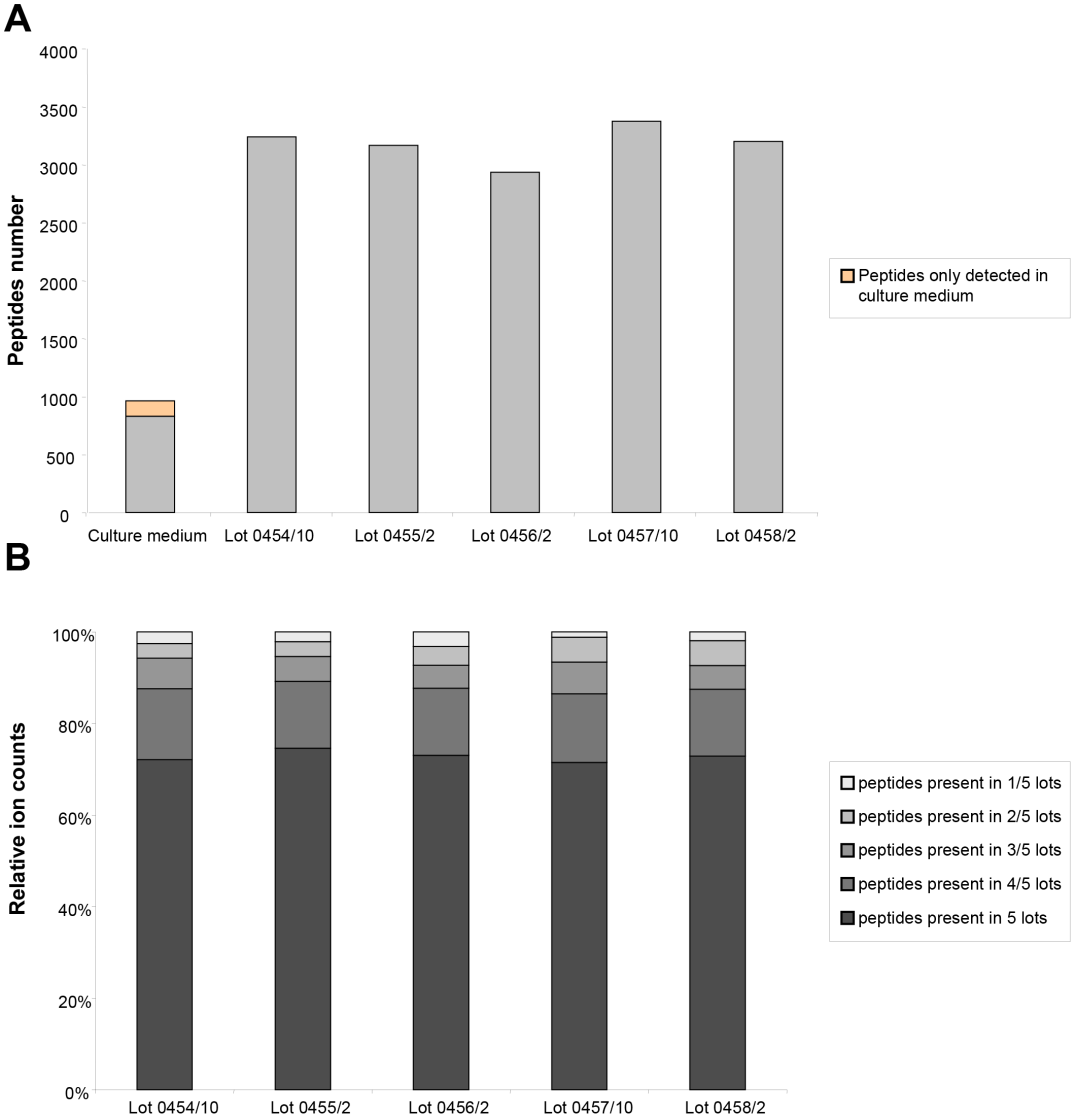
Similarity of different lots based on peptide content. Graphical representation of the presence of identical peptides. A) Shown is the absolute number of detected peptides in the 5 different Pro-Symbioflor lots and in the culture medium and B) peptide abundance based on relative ion counts of each lot.

As all five lots are derived from the same set of strains and were produced under identical conditions, similarity between the data in the 5 samples should be high, and distinctly different from the data obtained when analyzing medium only. The overall similarity of the 5 samples and the difference with medium only can be clearly observed by visual inspection of the contourplots (**Figure 1**). The similarity is also observed when analyzing the number and abundance of detected peptides in the different lots. In the bacterial lysates 2939 to 3381 different peptides were detected, while approximately 3 times less different peptides (964) were detected in culture medium (**Figure 2** **A**). 132 peptides were exclusively detected in culture medium suggesting transformation of these peptides by the bacteria. When looking at the relative abundance of the peptides, the similarity between the lots is striking as well. The abundance of peptides detected in all 5 lots (1391 peptides) represent 71–75% of the total content (based on ion counting) (**Table 2** and **Figure 2** **B**). If the absence of a single peptide in one of the 5 lots is accepted, an additional 894 peptides are present in four of the five lots. These peptides represent a total of 87–89% of all observed peptides, which suggests high consistency in all samples. Peptides which occur only sporadically (in one or two lots) represent only 5–7% of the total polypeptide content.

**Table 2:** Percentage of identical peptides present in a lot (expressed as the percentage of the total signal intensity in the lot) compared to the other lots.

Lot ID	0454/10	0455/2	0456/2	0457/10	0458/2	Culture medium	Total PPs*
**all 5** lots	72% (84%)	75% (84%)	73% (88%)	71% (83%)	73% (88%)	51% (68%)	1391
**4 of 5** lots	15% (12%)	15% (12%)	15% (9%)	15% (11%)	15% (9%)	12% (16%)	894
**3 of 5** lots	7% (2%)	5% (2%)	5% (2%)	7% (3%)	5% (3%)	11% (7%)	804
**2 of 5** lots	3% (1%)	3% (2%)	4% (1%)	5% (1%)	5% (1%)	8% (4%)	873
**1 of 5** lots	2% (1%)	2% (0%)	3% (0%)	1% (0%)	2% (0%)	5% (4%)	1255
only in **culture medium**	0% (0%)	0% (0%)	0% (0%)	0% (0%)	0% (0%)	14% (1%)	132

The percentage of sequenced peptides (expressed as the percentage of the total signal intensity) is given between brackets. *PPs, polypeptides.

Overall these data show, using a technique that allows grasping thousands of molecular features in a routine manner, high similarity of the 5 different lots.

### Sequencing of bacterial lysate peptides

**Figure 3:**
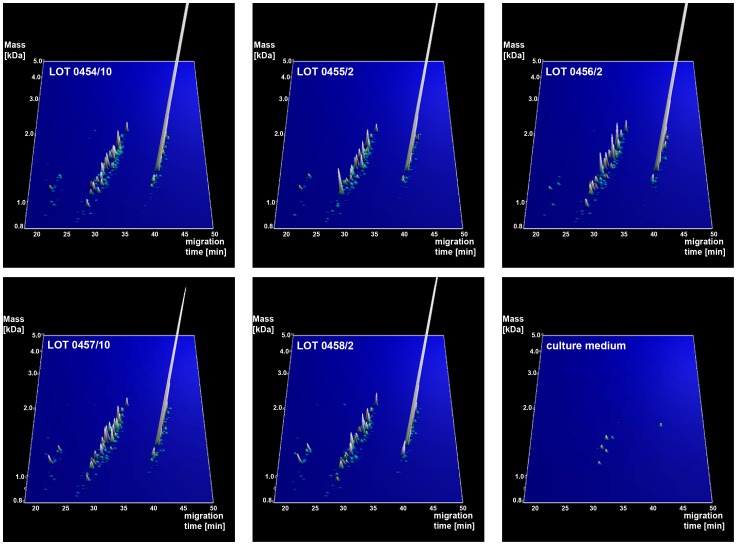
Expression profiles of sequenced bacterial peptides. Contourplots of the sequenced polypeptides originating from *E. coli* and *E. faecalis* in the 5 lots of Pro-Symbioflor and culture medium. To improve visualization of the peptides in this contourplot, the abundance of the peptides was artificially amplified with a factor 3 in relation to Figure 1. The 9 signals shown in culture medium are false positive hits by sequencing via LC-MS/MS. Given that 390 bacterial peptides were identified we obtained an acceptable false discovery rate of below 5%.

**Figure 4:**
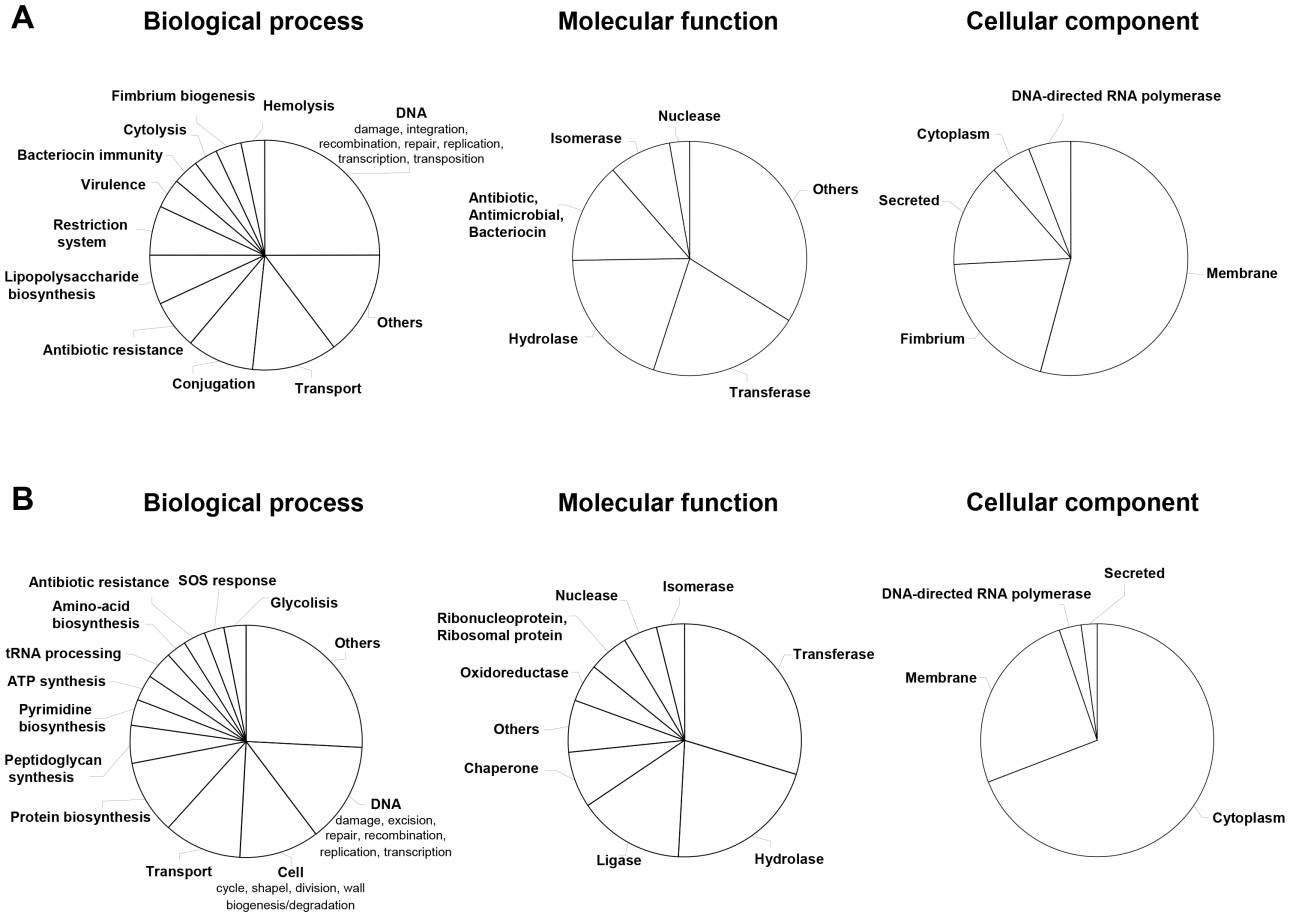
Ontology based characterization of the peptides identified by CE/MS. Based on the Uniprot identifiers the involvement of a protein (defined by one or more CE/MS peptides) in a biological process, molecular function and cellular component was determined. The pie-charts show the percentage of proteins in each group. Only classes represented by 4 or more proteins are shown. Classes for which we identified less than 4 proteins are indicated and grouped as “others”. A) Classification for *E. coli* and B) classification for *E. faecalis*.

To obtain additional information on the observed peptides and link the peptides to the different bacterial strains or to medium and potentially lay the groundwork for the identification of specific probiotic peptides, MS/MS sequencing was performed. The search for *E. coli* and *E. faecalis* entries in the lots resulted in identification of 517 peptides originating from *E. faecalis* and 406 originating from *E. coli*. These sequences (*E. coli* and *E. faecalis*) were matched to the CE/MS data profiles and resulted in identification of amino acid sequence of 390 CE/MS-detected peptides (**Table S1** and **Figure 3**). Ontology-based analysis of these 390 peptides showed that the majority of the identified cellular components identified for *E. coli* originated from the cell membrane and the fimbrium (**Figure 4**). In contrast, the major cellular components identified for *E. faecalis* originated from the cytoplasm. However, definition of the peptide-content based on molecular function showed in both strains enrichment for hydrolases and transferases. Processes involving DNA (i.e. damage, integration, recombination etc…) were the top class when considering biological processes in both strains.

**Figure 5:**
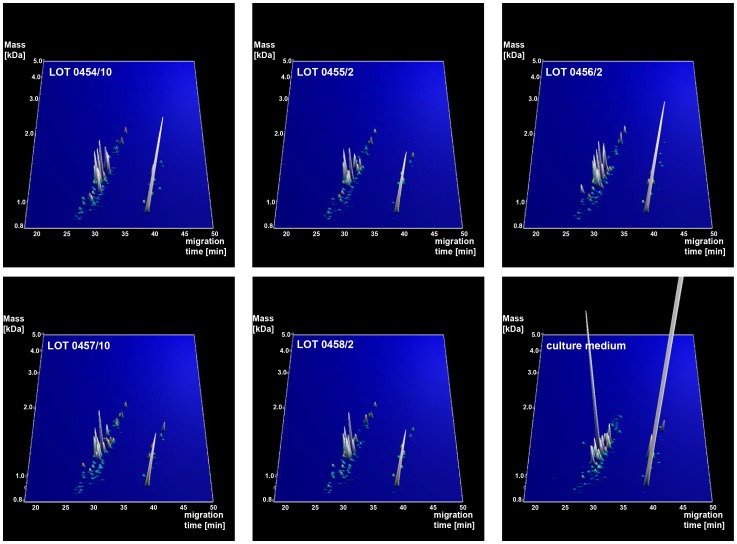
Expression profiles of peptides originating from the medium. Contourplots of the sequenced polypeptides originating from the culture medium only. To improve visualization of the peptides in this contourplot, the abundance of the peptides was artificially amplified with a factor 3 in relation to figure 1.

In culture medium 933 different yeast, 199 casein and 88 fragments from soybean could be identified in the MS/MS experiments confirming the composition of the culture medium (**Table 1**). Matching of the sequenced peptides of the medium to CE/MS detected peptides led to the identification of 175 CE/MS detected peptides (**Table S1** and **Figure 5**).

## Discussion

Quality control of inactivated bacterial lysates has concentrated on the control of the functional characteristics, like the quantification of expressed genes [31]. So far no targeted QC procedures concerning the identity of the strains for bacterial lysates are available after inactivation. Especially no cultural methods can be applied for classical identification and quantification. Only molecular methods are applicable. However, the complexity of bacterial lysates is high and therefore QC of the lysates is best performed with multidimensional techniques allowing the analysis of a maximum of molecular features at one time. CE/MS has been largely used for the definition of diagnostic and prognostic biofluid biomarkers of different diseases [23], [29], [32]–[35] and has been shown to be highly reproducible with a short turnaround time [24]. For this reason we decided to study whether CE/MS could be used for QC control of probiotic bacterial lysates. For QC in CE/MS analysis for clinical use, we 1) developed standard operating procedures for reproducible sample preparation [36]; 2) developed specific software solutions for efficient and reproducible data processing [37] and 3) identified internal calibrants that enable comparison of the datasets [28]. These quality control steps were applied to the bacterial lysates in the current study to minimize the variability and to achieve high reproducibility and can be used in future studies. Using CE/MS we showed high similarity between 5 different lots of the Pro-Symbioflor formulation on the peptidome level and identified, using peptide sequence analysis, the presence of proteins typical for the two strains included in the formulation. Clear differences between the peptide content of the Pro-Symbioflor formulation and the medium used for cultivation of the bacteria could also be observed.

The CE/MS approach provides evidence for Pro-Symbioflor lot similarity and content on several levels. On the total peptide level, including peptides with known and unknown sequence and where peptides are annotated with a unique tag comprised of mass and CE migration time, a pool of >5000 peptides can be used to obtain a peptide profile. Based on these peptide profiles, consisting of 2000–3000 peptides per lot, high similarity between the lots could be determined (**Table 2**). Sequence information of the peptides allows clear cut identification of the species present in the sample. The sequenced peptides clearly showed the presence of fragments of the two bacterial strains with comparable ratios between the different strains in the different lots.

Ontology analysis showed that the majority of the CE/MS-detected peptides of *E. coli* were identified as cell wall components which might have a role in activating the innate immune system, as has been shown for *Staphylococcus aureus* [38]. Parts of the cell wall of bacteria have also been successfully applied in atopic dermatitis [39]. These bacterial lysate peptides as a whole are recognised as highly conserved molecular patterns by the innate immune system as microbe associated molecular pattern (MAMP) [39], [40].

Upon matching of the sequenced peptides to the CE/MS peptides, nine sequences originating from either *E. coli* or *E. faecalis* were also found in the medium sample (see **Figure 3**). pBLAST analysis showed that all 9 peptides belonged with 100% homology to the bacterial sequences. Therefore, these 9 sequences were most likely not correctly matched to the CE/MS-detected peptides within the allowed mass and migration time deviation. This suggests that for a reduced number of peptides (2.3%), the resolution of CE/MS is not high enough to differentiate between two peptides with very close mass and CE migration time.

Sequence analysis identified with high confidence the presence of 88 peptide sequences that were fragments of soybean proteins (OMSSA Search, **Table 1**). This shows the ability of the CE/MS-based analysis to identify even hydrolysates in complex samples such as bacterial lysates.

Finally, CE/MS based detection of peptides originating from the medium also gives information on the QC for the medium used for cultivation of the probiotic bacteria. Our method therefore seems suitable to be used as a QC tool for both quantity and quality in complex samples to comply with safety rules for probiotics as required e.g. by EFSA through the QPS system [19].

Although, CE/MS has been extensively used for the discovery and validation of disease in biofluids this is, to our knowledge, the first study showing the suitability of CE/MS in quality control of complex probiotic formulations. CE/MS has been proposed in the QC of biopharmaceuticals (*e.g.* production of recombinant human interferon-β [41], detection of enantiomeric impurities (CE-MS/MS) [42], purity of drugs such as carbachol, lidocaine and proguanil [43], cosmetic [44]) or even at traditional Chinese medicines [45], but never for highly complex formulations such as inactivated probiotics [46].

In conclusion, our studies on Pro-Symbioflor lysate suggest that CE/MS is a well suited tool for the QC of inactivated probiotics.

## Supporting Information

Table S1 CE/MS Analysis of lots. All detected peptides using CE/MS are listed. Additionally shown are matched MS/MS hits of peptides. From left to right: Internal peptide ID, mass in Dalton, CE migration time in minutes, signal intensity of culture medium sample, organism, amino acid sequence, description of motherprotein, accession number (uniprot), start and stop of sequence, intensity of lots, frequency of found peptides, mean intensity of 5 lots, standard deviation, coefficient of variation in%, sequencing information for peptides originated from bacteria: organism, amino acid sequence, protein name, accession number (uniprot) and start and stop of sequence.
(XLSX)
